# Continuous In-Line Chromium Coating Thickness Measurement Methodologies: An Investigation of Current and Potential Technology

**DOI:** 10.3390/s21103340

**Published:** 2021-05-11

**Authors:** Adam Jones, Leshan Uggalla, Kang Li, Yuanlong Fan, Ashley Willow, Christopher A. Mills, Nigel Copner

**Affiliations:** 1 Wireless and Optoelectronic Research and Innovation Centre, University of South Wales, Treforest CF37 1DL, UK; leshan.uggalla@southwales.ac.uk (L.U.); kang.li@southwales.ac.uk (K.L.); yuanlong.fan@southwales.ac.uk (Y.F.); nigel.copner@southwales.ac.uk (N.C.); 2 Tata Steel, Research and Development, Swansea Technology Centre, Swansea SA2 8PP, UK; Ashley.Brew@tatasteeleurope.com (A.W.); Christopher.Mills@tatasteeleurope.com (C.A.M.)

**Keywords:** coating thickness measurement, chromium, steel substrate, optical metrology

## Abstract

Coatings or films are applied to a substrate for several applications, such as waterproofing, corrosion resistance, adhesion performance, cosmetic effects, and optical coatings. When applying a coating to a substrate, it is vital to monitor the coating thickness during the coating process to achieve a product to the desired specification via real time production control. There are several different coating thickness measurement methods that can be used, either in-line or off-line, which can determine the coating thickness relative to the material of the coating and the substrate. In-line coating thickness measurement methods are often very difficult to design and implement due to the nature of the harsh environmental conditions of typical production processes and the speed at which the process is run. This paper addresses the current and novel coating thickness methodologies for application to chromium coatings on a ferro-magnetic steel substrate with their advantages and limitations regarding in-line measurement. The most common in-line coating thickness measurement method utilized within the steel packaging industry is the X-ray Fluorescence (XRF) method, but these systems can become costly when implemented for a wide packaging product and pose health and safety concerns due to its ionizing radiation. As technology advances, nanometer-scale coatings are becoming more common, and here three methods are highlighted, which have been used extensively in other industries (with several variants in their design) which can potentially measure coatings of nanometer thickness in a production line, precisely, safely, and do so in a non-contact and non-destructive manner. These methods are optical reflectometry, ellipsometry and interferometry.

## 1. Introduction

### 1.1. Background of Functional Coatings, Applications, and Coating Thickness Methodologies

When applying a coating to a substrate, it is important to consider whether the measurement technique for coating thickness is sensitive in the thickness range required by the coating specification, as the thickness of a coating is one of the key variables in determining the effectiveness for its given function [1]. Coating weight, and hence thickness specifications, is chosen for optimum performance with regards to a particular application and it is common that under-coating and over-coating can both lead to negative effects [2]. Coatings are typically important for waterproofing applications [3], corrosion resistance [4,5], adhesion performance [6,7], aesthetic effects [8], painting applications, and several more needs. There are many different methodologies for monitoring coating thickness and each method is usually specific for an application dependent on the substrate and coating materials. There are several reported methodologies for thin film characterization for application in the photonics industry [9], metal finishing industry [10,11], medical industry [12,13,14], and semi-conductor industry [15,16]. The successful monitoring of coating thickness can produce results such as creating a product to the intended specification for the customer, cost savings resultant from the prevention of material wastage, and a greater yield output from the implementation of in-line control.

Coating thickness test methods are split into two main categories, destructive and non-destructive measurement [10]. There are also other factors to consider when applying a proposed method to a fast-paced production setting with a harsh environment. There are contact and non-contact coating thickness measurement methods which can be used for specific coating and substrate applications. A contact method for this application would be unfeasible, as the production line is continuous in nature, and these methods may cause damage to the product or even the production process. The need for high-speed measurement rules out several other methods such as X-ray Photoelectron Spectroscopy (XPS) and Transmission Electron Microscopy (TEM) [17]. Most accurate methods, such as XPS and TEM, require sample preparation [17], making them unfeasible for in-line measurement. Another requirement for this application is that the measurement method must have the ability to measure coating thicknesses under 50 nanometers in thickness. Although there are other important factors to consider for the measurement methods, these factors have been considered a priority for this application.

### 1.2. Background of Electrolytic Chromium Coated Steel (ECCS)/Tin Free Steel (TFS)

**Figure 1:**
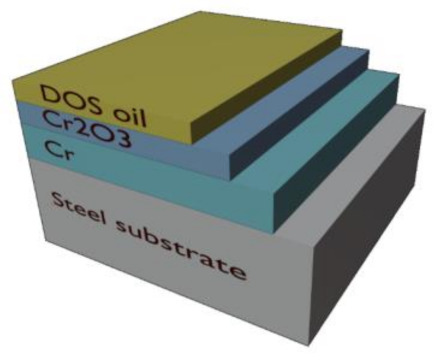
On-line ECCS layer structure for packaging steel. ECCS comprises a steel substrate, a metallic chromium layer, a chromium oxide layer and a Dioctyl Sebacate (DOS) oil layer. ECCS was developed in the 1980s for the packaging market, providing cost savings in comparison to tinplated packaging products as ECCS requires a much thinner coating to provide similar corrosion protection [20]. The steel substrate for this application is typically less than 0.5 mm in thickness and varies with customer specifications. The total chromium layer for this application (summation of both the metallic and oxide layers) has a maximum thickness of 50 nm and again varies with customer specifications, but the thickness of these coatings must also comply to European standards, EN 10202:2001, to ensure product quality requirements [21].

This review emerges from a requirement to measure chromium coatings on a steel substrate passing through a production line running at full speed, which can be more than 6 m s^−1^. For this application, metallic chromium (Cr) is electroplated onto both the top and bottom surfaces of a stainless-steel substrate in a series of chromium baths, this layer then passivates to form a chromium oxide (Cr_2_O_3_) layer. This product is widely known as ECCS or TFS, and its primary function is for the steel packaging industry. ECCS is used in many applications such as food and beverage containers, personal hygiene products, industrial packaging products and paint containers, as well as containers for gift products [18]. Steel packaging is highly recyclable and offers a long shelf-life which makes it a highly sustainable packaging solution, both for the present and the future [19]. Figure 1 illustrates the typical coating structure of ECCS.

### 1.3. Developments and Challenges in ECCS/TFS

The traditional ECCS manufacturing process utilizes Cr (VI) compounds in the passivation process of the steel. These Cr (VI) compounds are highly oxidizing substances which are easily reduced to Cr (III) compounds [18]. Unfortunately, with Cr (VI) compounds, there have been several studies that indicate their high toxicity compared to Cr (III) compounds in humans and animals, causing allergenicity and carcinogenicity through ingestion, dermal contact, and inhalation [22]. High concentrations of Cr (VI) have been found in US tap water, which may be resultant from the discharge of steel, pulp, metal-plating, and leather-tanning facilities, as well as through erosion of soil and rock [23,24,25]. REACH (Registration, Evaluation, Authorization and Restriction of Chemicals) legislation require the withdrawal of the use of chromium (VI) compounds due to safety concerns [18], which requires the steel packaging industry to develop new substrate variants [20]. One of the most recent developments in this area is Trivalent Chromium Coating Technology (TCCT), which is based on a chromium (III) process [6,26].

### 1.4. Continuous In-Line Chromium Coating Thickness Measurement

In addition to the ECCS application, thin chromium and chromium oxide films have been studied in several other applications, such as electrode materials for solar energy conversion and electrochromic windows [27], masking for lithography [28], and improvement of adhesion to transparent substrates [29]. The overall aim of this research is to investigate current and potentially novel in-line coating thickness measurement methods, applicable to chromium coatings on a steel substrate within a steel production setting, which could potentially be used for other applications highlighted above, or even other thin film coatings, with the following requirements:

- Non-destructive;
- Non-contact;
- Ability to measure nanometer coating thickness;
- High speed measurement for a continuous production setting;
- Feasibility for measuring multilayer coating structures;
- Feasibility of the methodologies in terms of the substrate material and coating material.

This research has been driven by a need for a new in-line chromium coating thickness measurement method for ECCS and TCCT coatings for packaging steels. The most common in-line coating thickness test methods for this application are based on X-ray Fluorescence (XRF) methods [30] however, these systems can become extremely costly, especially when implemented for a wide ECCS product, and impose health considerations, as they involve ionizing radiation. In terms of chromium thickness measurement, there is very little literature for an in-line measurement technique, except from similar application cases implementing several variants of ellipsometry [31,32,33,34,35,36]. Here, we examine the potential methods for in-line coating thickness measurement methods for ECCS and TCCT [33], and includes not only the measurement of the thin metallic chromium layer, but also the chromium oxide and DOS oil layers [31,32]. Therefore, this article aims to review potential methods for the in-line measurement of nanometrically thin chromium layers on a steel substrate for packaging applications, which could potentially be implemented into other industries. This review will briefly cover the operating principles of traditional, offline, and potentially novel in-line coating thickness measurement methods and summarize the advantages and limitations of these methodologies. Development of an accurate in-line coating measurement system would determine the precise coating thickness, removing the need for over-plating (excess electrical current), and consequently providing both cost and environmental benefits.

## 2. Traditional Coating Thickness Test Methods

Traditional coating thickness test methods are well known techniques that have been used previously and currently to measure coating thickness in several applications.

### 2.1. Coulometry

**Figure 2:**
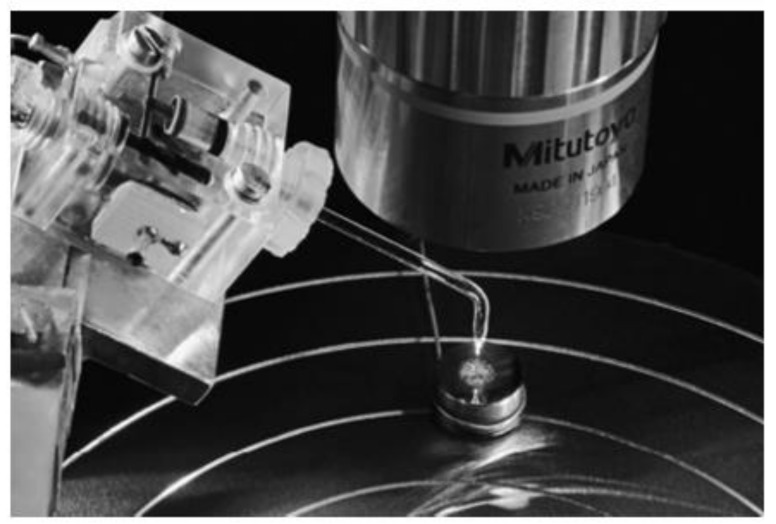
Photograph of an electrochemical cell used in coulometry. The tip of the cell has direct action with the coating structure, with a crater formed on dissolution on the sample. (Reprinted from [37], Copyright © 2011 John Wiley & Sons, Ltd.).

Coulometry is a destructive method in which the coating weight is measured by stripping the coating off the substrate and a mass per unit area relative to the coating thickness is calculated [10,37,38]. This method uses the reverse method of the electroplating process used to deposit coatings and the measurement of the coating is calculated using an electrolysis cell which contains electrolyte specifically designed for stripping the intended coating [38]. Constant current runs through the test cell and strips the coating (as the coating surface acts as the anode). As the current and surface area remains constant, the coating thickness can be calculated relevant to the time taken to strip the coating off the substrate. This method is very effective for electrically conductive coatings on a conductive substrate [10]. Schneider et al. [37], conducted a study to compare coulometry and optical reflectometry for thickness determination on titanium oxide films, which lead to the potential further study of an in situ spectroelectrochemical cell for this particular application This method has high measurement accuracy, is a low-cost system, can measure multilayer coatings and can be useful for a wide range of offline coating thickness applications. As this is a destructive process, this method is not feasible for in-line measurement. Figure 2 illustrates an electrochemical cell used in coulometry. 

### 2.2. Beta Particle-Backscattering

**Figure 3:**
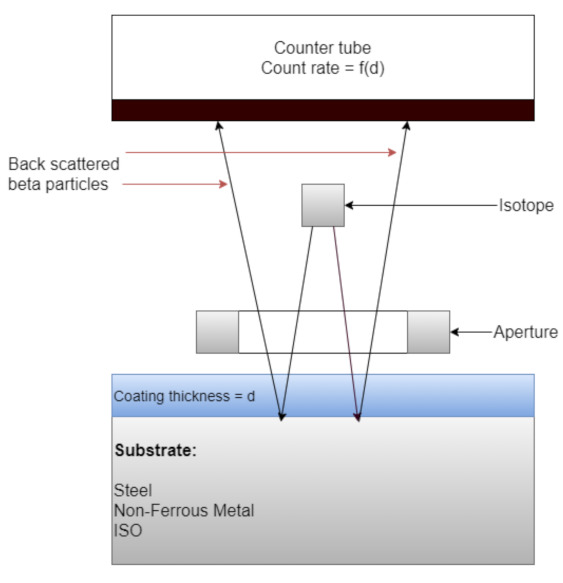
Operating principle of Beta-backscatter method. (Adapted from [10], Copyright © 2001 Published by Elsevier Inc.).

For the Beta particle backscattering method, the sample under test is exposed to beta particles from a beta emitting isotope. Particles are directed though an aperture onto the sample with the coating to be measured. A percentage of these particles are backscattered back through an aperture into a Geiger–Muller tube [10]. Upon interaction of the backscattered particles with the gas within this tube, an ionization event occurs which is detected by the tube electrodes, held at a high potential difference, forming an electrical pulse which is then electronically counted. Materials of low atomic number backscatter at a lower rate than materials with a high atomic number. The change in the rate of electrons backscattered is a measure of the coating thickness. However, for this method to work effectively, the atomic number difference between the coating and the substrate must differ by at least 5 atomic units. Chromium’s elemental atomic number is 24, whereas steel, predominantly iron, has an atomic number of 26, so the atomic number difference between these elements is only 2, rendering this method not feasible for this measurement application. Figure 3 illustrates the operating principle of the beta-backscatter method.

### 2.3. Eddy Current

**Figure 4:**
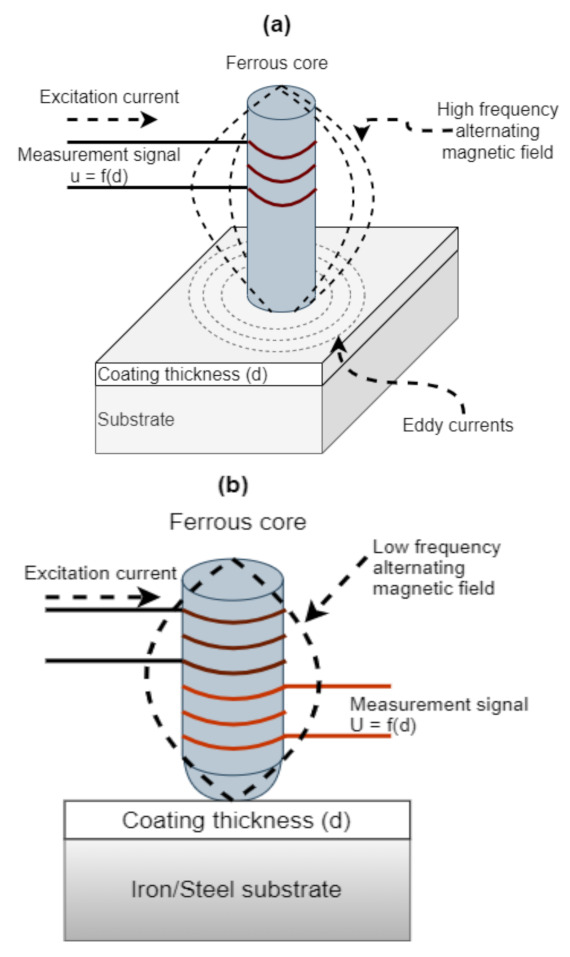
(**a**) Eddy current operating principle. (Adapted from [10], Copyright © 2001 Published by Elsevier Inc.). (**b**) Magnetic induction operating principle. (Adapted from [10], Copyright © 2001 Published by Elsevier Inc.).

The eddy current method uses a probe which contains a current carrying coil which is driven by a high frequency oscillator to generate an AC high frequency magnetic field. When the probe comes into proximity with the sample, eddy currents are generated at the sample, which alter in amplitude and phase to the original magnetic field. This difference generates an impedance signal between the excitation coil and the sample which is related to the coating thickness. This impedance change is dependent on the distance between the probe and the conductive substrate material. There are two main alternatives to this method, measuring the phase or the amplitude of the impedance changes [10]. Eddy current techniques are widely available commercially and offer micron coating thickness resolution. There is literature available for this technique, including the development of Pulsed Eddy Current (PEC) techniques [39] and research into varying probe constructions [40]. Eddy current measurements can be made on nonconductive coatings on nonferrous conductive substrates, nonferrous conductive coatings on nonconductive substrates and nonferrous metal coatings on nonferrous metals, rendering this method not feasible for this application, as the steel substrate is ferromagnetic. Figure 4a illustrates the operating principle of the eddy current method for coating thickness evaluation. 

### 2.4. Magnetic Induction

This method consists of a measurement probe containing a low frequency AC coil which is placed on the sample surface. By bringing a ferromagnetic substrate into the magnetic field, the core magnetic flux density is changed and is captured by a secondary sensing coil. The difference between the contact point at the sample and the distance to the substrate would equal the coating thickness. The typical time for a measurement ranges between 50 and 100 ms [10], which has potential for in-line measurement; however, this method generally requires contact with the sample, and has only micron level thickness determination. The magnetic induction method is used for measuring thickness of nonferrous coatings on ferro-magnetic substrates, thickness of magnetic coatings on non-magnetic substrates and thickness of magnetic coatings on magnetic substrates if the permeabilities of the coating and substrate are different and constant. Both Petrilli [10] and Hinken et al. [41] express the application of the eddy current and magnetic induction methods unfeasible for this application (ferromagnetic stainless-steel substrate) due to the limitations of the substrate/coating material combinations along with the thickness limitations, rendering this method not feasible. Figure 4b illustrates the operating principle of the magnetic induction method for coating thickness evaluation. 

### 2.5. X-ray Fluorescence (XRF)

XRF is a measurement method which can be used to determine the compositional analysis of solids, liquids, powders, and can also be used to determine the thickness of coatings. This method can measure elements from Beryllium (Be) to Uranium (U) at sub-ppm levels. Elements with higher atomic numbers have better detection than lighter elements. This method is fast, accurate and non-destructive and can measure in a continuous production process for in-line measurement. The measurement time of a sample can range between seconds and minutes [30] and is based on the number of elements to be examined within a sample and the required accuracy. As the sample is irradiated with X-ray excitation, the sample will emit fluorescent radiation with discrete energies that are related to the elements of the sample. These energies differ by color, and this determines the different elements of the sample which provides qualitative analysis of the sample. Measuring the intensity of this fluorescent radiation can determine the quantity of each element present within the sample, which provides quantitative analysis. Fluorescent radiation from a sample occurs when the incident source expels an electron from an atom with a higher energy level. This produces a vacancy in the inner electron orbit, causing instability within the atom. The atom naturally wants to restore stability by transferring an electron from the outer to the inner orbit. This process emits fluorescent radiation in the form of a photon by the energy required for this orbit transfer. XRF methods are split into two main groups, Energy dispersive (ED-XRF) and Wavelength dispersive (WD-XRF). ED-XRF can measure from Sodium (Na) to Uranium (U). This method is comprised of detectors that measure the different energies of the characteristic radiation from the sample. WD-XRF can measure from Beryllium (Be) to Uranium (U). This method uses an analysis crystal that disperse the differing energies from the irradiated sample. This radiation is diffracted off the crystal in differing directions like a visible spectrum prism. ED-XRF systems feature fast measurement times, which is commonly used for in-line coating thickness measurement. Both ED-XRF systems and WD-XRF systems are currently being used in this research application to measure chromium coating thickness in-line and offline; however, there have been issues regarding the in-line system accuracy. These systems are very costly, and to implement an ED-XRF system for a wide product in a continuously fast-moving production environment, can become extremely complex. There are many applications for this measurement method [10,11,30,41,42], although this research originates from a need to investigate another potential in-line method, rendering this method not feasible for this application.

### 2.6. X-ray Reflectometry (XRR)

XRR operates on a similar theory as reflectance spectroscopy [43]. This reflectance measurement comprises of an incident ray, which reflects off the different interfaces at the sample, which form interference patterns through a range of incident angles. The incident ray in XRR consists of a beam of X-ray photons. The incident angle of this ray can be varied to give variations in the amplitude of the reflected beam, which can provide information on the coating and substrate. The incident ray will reflect or transmit through the sample at differing optical path lengths and amplitudes. Varying the incident angle can supply information on the sample, and to vary the incident angle, a Goniometer [10] can be used with reference to the source and detector, this slow process can take between 30 and 60 min. There are advancements within focused beam optics that can decrease this measurement time from 1 to 10 s. Typical additions to the system to increase performance are the monochromator, entrance aperture and collimators. This method is a very costly solution but does feature all the requirements needed for this application. Research has already been conducted with this method, investigating nanometer thick chromium coating thickness on a silicon substrate [44], which provided positive results when comparing with SEM methodologies. This method looks highly feasible for this application, however, to implement this system for a full product width would not provide the detection times needed for a continuous production line, and therefore it can be considered not feasible for this application.

### 2.7. Ultrasonic Detection

The ultrasonic method is a non-destructive technology (NDT) for the coating thickness measurement of primarily wood, concrete, and plastic applications. This method comprises of a single element transducer and numerical techniques to filter sound wave echoes from a sample. Current instruments on the market typically need to apply contact to the sample. The principle of operation is that a probe is placed upon the sample surface, a sound wave is propagated through the sample which results in differing vibrations at each layer interval. These vibrations are received at the probe source and numerical calculation is conducted to investigate the appropriate thickness of each layer. Novel advances to this method include high frequency scanning acoustic microscopy to measure sprayed coating thickness, non-destructively [45]. This method has been reported not ideal for thin metallic coatings or metallic substrates and conventional gauges require contact with the sample, which renders this method not feasible for continuous in-line implementation [46].

### 2.8. Overview of Traditional Coating Thickness Methodologies

Table 1 summarizes the feasibility for in-line implementation for this research application. These methods are the most common and traditional coating thickness measurement methods, surveyed within the current literature. It can be noted that there are clearly several key variables to consider for these technologies, and the only traditional test method outlined that could be used is the XRF method (in this case, ED-XRF for the fast measurement response). This test method is currently implemented in-line for this application and has several disadvantages, such as being a costly system to implement for a full product width, the measurement of total chromium only (unable to differentiate between oxide and metallic components) and has reported inaccuracies when comparing with offline test methods. Therefore, novel methods were needed to be explored to address these disadvantages, to provide a potential complimentary system which could be low-cost and not utilizing ionization radiation for safer working practices. Seven traditional coating thickness measurement methodologies are reviewed for this steel packaging application within Table 1. These are Coulometry (A1), Beta-backscatter (A2), Eddy current (A3), Magnetic induction (A4), XRF (A5), XRR (A6), and Ultrasonic detection (A7). All the traditional methodologies highlighted are currently available commercially, however, the typical cost and pricing of most of the systems is provided on a case-by-case quotation. The eddy current, magnetic induction and ultrasonic methodologies are widely available on the current market for several coating thickness applications, and the price is dependent on the complexity of the application requirements. The thickness ranges, sampling rate, spatial resolution and measurement accuracy of each system are also dependent on their individual system design.

## 3. Offline Coating Thickness Test Methods

Offline measurement methodologies have been characterized as methods which do not possess detection times fast enough for continuous production settings, are destructive in nature, require contact with the sample under test, require sample preparation or also require vacuum sealed environments.

### 3.1. X-ray Photoelectron Spectroscopy (XPS)

XPS is a measurement method which can be used to quantitatively measure coating thickness by identifying elemental composition of a sample via the photoelectric effect. A sample under test is irradiated by a beam of X-ray radiation (photons) which excites an electron spectra resultant from the kinetic energy of the transmitted electrons. The typical measurement time of an XPS system differs dependent on the type of analysis performed, but the minimum analysis time is roughly 1 min, which renders this technique an offline measurement solution [17]. This method, however, is very accurate and can directly quantify coatings in nanometer scale thickness; however, this system is a very costly solution. Although this method requires sample preparation and requires a vacuum sealed environment, the measurement process is typically non-contact and non-destructive [17]. XPS studies have been conducted for monitor similar application areas, such as studying the surface chemistry of Zn-Al alloy coatings on steel [54] and studying the effects of passive films on stainless steel [55]. XPS is a well-known technique for investigating several different coatings, such as oxide thickness determination [56] and is also a method used currently for this research application to investigate the thicknesses of chrome oxide and metallic chromium layers, offline. Due to the cost and requirement for a vacuum environment, this technique is not feasible for in-line measurement.

### 3.2. Scanning Electron Microscopy (SEM)

**Figure 5:**
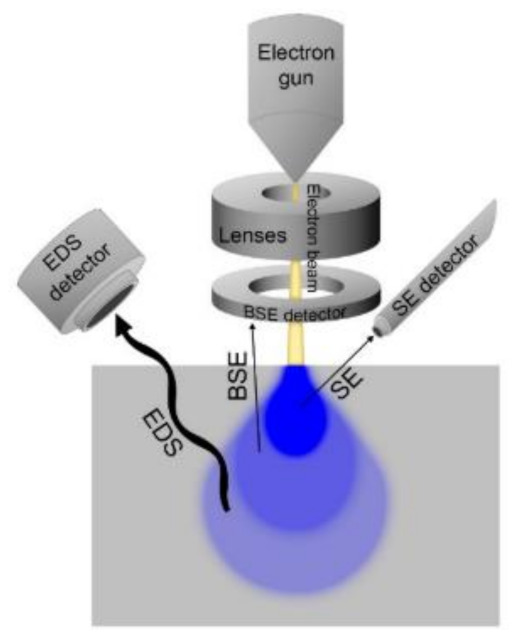
Operating scheme of SEM with SE, BSE, and EDS detection. (Reprinted from [26], CC BY 4.0 license).

Electron microscopes permit the observation of material composition at the nm and μm level. Electron microscopes are instruments which use a beam of electrons to examine an object on a magnified scale. This examination can provide information of the topography, morphology, composition, and crystallographic properties of a sample. A source of electrons is beamed over the surface of a sample. When the electrons penetrate the surface, several interactions occur that result in the emission of electrons or photons through the surface. These electrons can be detected, and an output is derived by modulating the brightness of a cathode ray tube [9] in terms of voltage. Many kinds of samples can be analyzed such as metals, ceramics, glass, hair, bones, and plastics. The main constraints of SEM are that the sample must be conductive (non-conductive materials must be carbon coated) and materials with a smaller atomic number than carbon cannot be detected. The vertical resolution of current SEM instruments ranges from between 0.6 and 1.5 nm [9], depending on the primary voltage. Giurlani et al., provide a comprehensive review on SEM technology, the advancement of SEM over time and the different detection methodologies used to create an SEM image, such as using secondary electrons, backscattered electrons or through a microanalysis map [26]. This method is considered not feasible for in-line measurement as it requires extensive sample preparation and highly controlled environmental conditions that are not suitable for in-line inspection. Figure 5 illustrates the operating principle of SEM.

### 3.3. Atomic Force Microscopy (AFM)

The field of Profilometry has a wide range of methods for investigating surface topographical analysis with sub-nanometer resolution. AFM measures the surface profile and film thickness of a sample through direct mechanical action. This method functions by directly measuring the deflection and oscillation of a flexible, microscopic cantilever tip caused by attractive and repulsive atomic forces when the tip is moved vertically in respect to the film surface. The AFM system uses a cantilever, tip, laser source, and a scanner. The laser source focuses a beam of light onto the rear face of the cantilever tip and the photodetector measures the intensity of the beam off the tip. The system may also use a voltmeter or a waveform generator to measure the oscillation of the cantilever or drive the oscillation with a waveform. An AFM system may be used in two modes, a contact mode in where the tip remains in contact with the sample during testing and a tapping mode in where a spring force mechanism is implemented on the cantilever tip. This measurement method is highly accurate and allows for calculation of surface roughness, however, this method requires contact with the sample under test and the resolution depth is limited by its magnification range [9]. There are advancements within AFM which requires no contact with the sample under test [57], and there is an abundant amount of literature covering the different operating methodologies This method is considered **not** feasible for in-line measurement due to the need for sample preparation and the detection speed to determine coating thickness. AFM has been used in several thickness measurement applications, such applications include the thickness measurement of graphene onto oxidized Si wafers [58], thin metal films on silicon substrates and polymer films on silicon substrates [59].

### 3.4. Glow Discharge Optical Emission Spectroscopy (GDOES)

This technique comprises of an optical emission spectrometer (OES) coupled with glow discharge (GD), and allows for the monitoring of both the surface and depth profiling of the elemental composition of solid materials, with high sensitivity. This technique is destructive in nature and involves sputtering an area of a sample with GD plasma, whilst observing optical emission. This technique has vertical resolution at the nm level and can identify which elements are present within a sample, quantify the concentration of elements in a sample, and in this research application case, measure coating thickness within a sample. GDOES has been used to measure coating thickness and elemental composition in several applications [60,61,62]; however, as this technique is destructive in nature, this method is assessed as not feasible for this application.

### 3.5. Overview of Offline Coating Thickness Methodologies

Table 2 provides a summary of offline coating thickness measurement methodologies. These methods have been considered not feasible for in-line measurement due to their limitations outlined and have been briefly researched to demonstrate and convey why they are considered unfeasible. All the offline measurement systems outlined have high accuracy and can determine nanometer scale thickness, but all methods require sample preparation. In terms of the detection speeds listed above, these are relevant for this application (coatings under 100 nm in thickness). From the literature, each of these methods and method variants have advanced greatly over the past two decades, with results in faster detection speeds. Four offline coating thickness measurement methodologies are reviewed for this steel packaging application within Table 2. These are XPS (B1), SEM (B2), AFM (B3), and GDOES (B4). As noted from the review table for the offline coating methodologies, the thickness ranges and measurement accuracy have an increased vertical resolution over the traditional methodologies, at the expense of the increase in system cost. There are many commercial systems available for these techniques which have been surveyed and highlighted, and XPS technology is by far the most expensive (where systems can be in the cost excess of GBP 500,000). It must also be noted that all offline methodologies can determine the coating weight of multi-layer structures through direct measurement, at the expense of slow measurement speeds (typically within the range of minutes to hours).

## 4. Potential In-Line Coating Thickness Test Methods

In-line measurement methodologies have been characterized as methods which have the potential to measure real time, non-destructively and require no contact with the sample under test.

### 4.1. Thermoelectric Method with Magnetic Readout

The thermoelectric effect with magnetic readout takes in principle the Seebeck effect of bi-metallic materials coupled with a non-contact magnetic readout [41]. The Seebeck effect is mostly known for its application in thermocouples, an instrument that provides a voltage output approximating the applied heat at the junction of bi-metallic materials [67]. This method has been studied for Non-Destructive Testing (NDT) like the magnetic induction method, without contact with the sample [41,68] and is operated in the following steps:

- At the junction of dissimilar electrical conductors, a thermoelectric voltage is present when the junctions are at differing temperature.
- As this thermoelectric voltage would create a closed circuit regarding the coating and substrate, a current will flow from the hot junction to the cold junction.
- In turn, this electrical current will generate a magnetic field with a flux density that would extend to the outside of the material and into the air interface.

This method is largely dependent on the magnetic field strength that occurs at the air interface and the sensitivity of the magnetic sensor. The strength of the magnetic field can be influenced by the temperature gradient applied to the sample. A reverse process of this effect is known as the Peltier effect [41]. Applying the sample to an external magnetic field will induce currents and therefore voltages. This difference in voltage at the interface junctions will give rise to a change in temperature which could potentially be monitored with a thermoreflectance approach [69], but this technique will be discussed separately later. This method would provide non-destructive, non-contact analysis, and a high potential for in-line measurement. For nanometer scale measurement, it would be expected that the process heating phase would require a large differential to excite a magnetic field strong enough to detect with present magnetometers, based on the results from Hinken et al., experiments at the micrometer thickness ranges [41].

### 4.2. Terahertz Time Domain Spectroscopy (THz-TDS)

This method involves the use of high frequency radio waves (typically pulsed laser radiation) to reflect off the sample to provide information on the coating thickness [70]. A typical example of this method utilizes pulsed-echo terahertz thickness measurement by sending terahertz energy via a transceiver and to reflect off a conductive material substrate back to the transceiver. The terahertz transceiver is separated from the sample by an air path which makes this method non-contact and non-destructive in nature [71]. There is little research regarding this method in terms of nanometer scale resolution; however, this technique looks like a promising method for continuous in-line implementation [72,73]. The operating principle of this method involves the time domain analysis of the delay between the received echoes from the top surface of the coating and the top surface of the substrate. This method has a very similar operating principle to the optical reflectometry method; however, the detection and analysis of this method is not a function of optical intensity as in reflectometry, but as a function of the difference in time. The time delay between the coating surface echo (FS) and the substrate surface echo (BS) is directly affected by the thickness variation of the coating [73].

### 4.3. Optical Reflectometry

**Figure 6:**
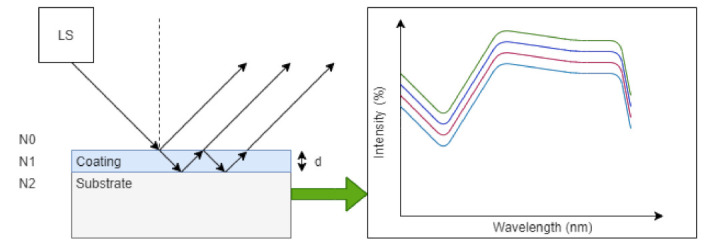
Principle of optical reflectometry. A light source (LS) illuminates the sample of interest and as a result, there are several reflections returned to the detector due to layer interference. As the layer thickness is increased or decreased, dependent on the optical properties of the layer structure, the resultant reflection spectrum can determine coating thickness as a function of reflection intensity.

Optical reflectometry is a method which analyzes the reflectance spectra of a sample to measure characteristics such as optical constants and thin film thickness and is a well-known and powerful technique for measuring film thickness quickly in several industries [12,74,75,76,77]. There are extensive variants of this method, which are widely available for review in the literature, but the key operating principle of this methodology is the illumination of a sample with an optical light source, detection of the reflected intensity and fitting this intensity as a function of coating thickness [76]. Most technologies utilizing this method measure a range of wavelengths to calculate the film thickness, and one of the key limitations to this measurement method in the past is that the technique is only able to measure one point at a time [76]. Variants of this method have included volumetric detection using Charge Coupled Devices (CCD) to monitor an area of a sample, simultaneously, with the disadvantage of decreased detection speeds, but there is literature available into techniques which can reduce these detection times, such as direct phase extraction techniques [78]. Reflectometry methods usually operate at normal incident angles for measurement; however, varying incident angles can be used which again increases the system complexity in terms of monitoring both parallel and perpendicular polarizations, based on the Fresnel equations [79]. This method also consists of a form of self-interferometry, in where there are several internal reflections between the coating and substrate interfaces. These coating and substrate interfaces are often modelled using the Fresnel reflection coefficients to determine the reflected intensity present at the optical detector, however, for several layers these calculations become complex, and researchers tend to model these interfaces using Transfer Matrix Methodologies (TMM) [80]. The main operating principle for this method relies on reflection intensity, which is calculated by the ratio of the reflected intensity at the detector to the incident intensity. In terms of measurement limitations for this application, to measure metallic coatings, there is a requirement that the incident radiation can transmit through the coating to the substrate, and for chromium coatings, the calculated limitation through Beer’s law is roughly below 70 nm coating thickness. As this application intends to monitor thicknesses of less than 50 nm, this method has high feasibility [29]. This method has high speed capability, has the potential to measure the thickness ranges of the application area, is a non-contact and non-destructive approach which renders this method high feasibility for future research. There are several factors that must be considered taking this method forward, such as the surface roughness of the coating and the substrate, the coating uniformity and the production process parameters that could hinder measurement, most importantly, the vibration of the electroplated product running continuously through the production line. Figure 6 illustrates the operating principle of optical reflectometry for coating thickness evaluation. 

### 4.4. Optical Interferometry

**Figure 7:**
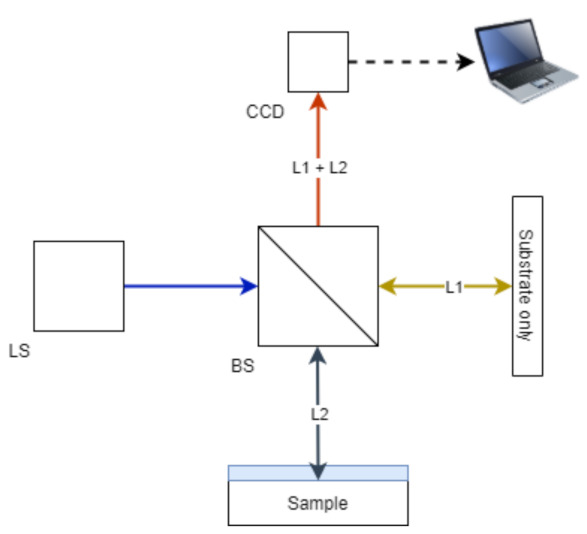
Principle of optical interferometry (Michelson setup) for coating thickness applications. A non-polarizing Beamsplitter (BS) separates a light source into two separate elements determined by the beam splitting ratio (typically 50:50). The transmitted light reflects from a reference sample (substrate only) at L1. The reflected light from the Beamsplitter irradiates the sample and undergoes varying self-interference dependent on the thickness of the coating outlined within the reflectometry method, coupled with L2. Both elements combine at the Beamsplitter and are then transmitted to a Charge Coupled Device (CCD). Signal processing is the conducted on the interference signal to evaluate coating thickness.

Interferometry is a very similar optical technique to the reflectometry method in that they both use the phenomena of optical reflection or transmission to gain an understanding of a sample under measurement. Where reflectometry measures the intensity of a reflected or transmitted spectrum and takes into consideration the self-interference of the layers of a sample, the interferometry method takes into consideration the optical path difference in relation to a single light source between a sample under test and a reference mirror via a beam splitter [81]. As the optical path difference will change relative to the reference mirror and the sample as a function of coating thickness, differing phases based on the optical path difference will provide constructive or destructive interference for the output signal. There are many variants of optical interferometry, such as using differing light sources such as white light or singular wavelength [16,82], differing optical path layouts [83,84], and differing detection elements [85,86]. There are many interferometric solutions for the measurement of thin film thickness which operate on the principle of interference but utilize different components within the system for different applications. An interferometric and reflectometric method which may be suitable for this project’s application was reported to be non-destructive, non-contact and has high-speed data acquisition, has published results within the nanometer thickness range in a microelectronics production setting [81]. Typically, interferometry is limited by a factor of the incident wavelength of light, however, the proposed system [81] utilizes Fast Fourier Transforms for the surface topography coupled with a reflectometric approach which has highly promising continuous in-line implementation. The setup proposed is basically a Michelson interferometer with two arms, one to generate the reference wave from a flat mirror and one to generate the reflection from the sample of interest. Two similar objective lenses are added into the system to adjust the lateral measuring magnification. The thickness ranges for this method have been reported down to a single nanometer [87], but like reflectometry, this setup is limited to metallic layers within the optical transparency region, which renders interferometry highly promising for this application. Figure 7 illustrates the operating principle of optical interferometry for coating thickness evaluation. 

### 4.5. Optical Ellipsometry

**Figure 8:**
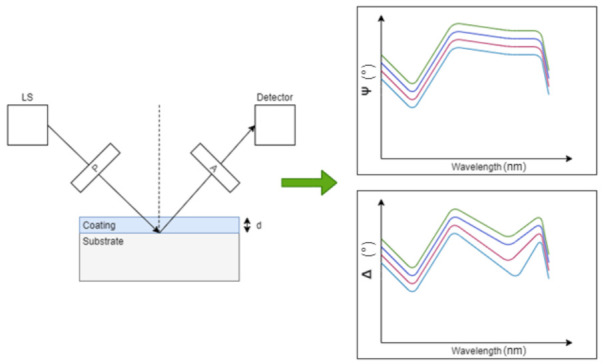
Principle of optical reflection ellipsometry for coating thickness applications. A polarizer (P) linearly polarizes a light source into a known polarization state at incidence of a sample. As light interacts with the sample, a change of polarization for both parallel and perpendicular polarizations will occur, dependent on the sample properties and coating thickness. An analyzer (A) is used to detect the polarization state of both polarizations, which is then fed to a detection element for further analysis. A ratio of both the parallel and perpendicular polarization determines the amplitude and phase difference generated from the sample of interest, which can then be compared to pre-determined optical model to measure several parameters, such as coating thickness, coating uniformity, and optical constants [95].

Ellipsometry is an optical measurement method using light reflection or transmission of a sample. This method is very similar to the reflectometry method; however, ellipsometry also measures the change in polarization regarding reflection or transmission on a sample. This method can be high speed in operation with the ability to measure nanometer scale coating thickness, giving rise to real time measurement [32]. This method measures two variables, ∆ and Ψ, where ∆ is the phase difference between parallel (p) and perpendicular planes (s) and Ψ is the amplitude ratio of parallel (p) and perpendicular (s) planes [88]. To put the method simply, a light source generates a beam of light that is transmitted through a linear polarizer and compensator to control the incident polarization of the light at the sample surface. Reflections occur at each interface within the sample at a given amplitude and phase. These reflections are analyzed and detected and can determine specific properties of each layer of the sample, whether it is to evaluate coating thickness, coating uniformity, surface roughness or even to determine the optical constants for the coating structure [89,90]. There are several variants of ellipsometry and its optical setup, such as using spectroscopic or singular wavelength light sources [91,92], using numerous passive optical components setups [93,94] and using imaging techniques [1]. This method is covered extensively in the literature for application for in-line coating thickness measurement in several industries, such as the measurement of thin film thickness of photovoltaic development on production lines [34], the monitoring of optical constants and layer thickness of organic photovoltaics in a roll-to-roll (r2r) production setting [35] and the evaluation of optical constants, layer thickness with nanometer precision and uniformity of organic electronic (OE) devices [36]. Extending in-line ellipsometry further for this steel packaging application, there are two publications in which researchers applied ellipsometry to extremely similar steel packaging applications to this research [31,33]. Firstly, Izumidate et al. [31] researched and developed an in-line ellipsometry system to measure the ultra-thin oil layer that is applied to the steel packaging product after the coating process, and to measure the hydrated chromium oxide layer that occurs through passivation after the metallic chromium layer has been electroplated onto the steel substrate. This research was reported to have been successfully implemented to measure these layers, continuously in-line with nanometer thickness ranges, which gives high potential for this application. However, the difference for this application is that the metallic chromium layer also needs to be measured in-line, which may have been a challenge as the metallic chromium thickness on their Tin Free Steel (TFS) product had reported millimeter thickness ranges, which would result in no optical transmission through the coating layer to the substrate interface. Secondly, Rischmueller et al. [33] conducted research utilizing the ellipsometry method for the inspection of future REACH (Registration, Evaluation, Authorization, and Restriction of Chemicals) compliant Trivalent Chromium Conversion Coatings (TCCC) on an aluminum substrate. This research article provided great depth on measuring this coating thickness within the optical transparency region of chromium (<70 nm), researching the most effective wavelength for their product with angular dependency and mitigation controls regarding the surface roughness resultant from the rolling process. It was reported [33] that this method could be implemented in-line, with a non-contact approach which is like this research application. Given the requirements of this application for nanometer chromium thickness measurement on steel with a measurable surface roughness, further research would need to be conducted into the feasibility of this method, however judging from the literature available, this method looks highly promising. Figure 8 illustrates the operating principle of optical ellipsometry for coating thickness evaluation. 

### 4.6. Stimulated Brillouin Scattering (SBS)

Stimulated Brillouin scattering is non-linear scattering which involves the artificial generation of acoustic phonons in a transparent medium. This type of scattering comes from light interaction with acoustic waves in a material and are generated by thermodynamic fluctuations [96]. This scattering method has been used in a spectroscopic nature to non-invasively provide general imaging solutions for applications in biology and materials science [97]. The SBS method utilizes two incident light waves at two differing frequencies, and the waves generated in the sample are the density variations (acoustic waves) that are resultant from the incident light waves which develop a beat frequency, which can then be monitored to provide information on the coating thickness. For this method to work for this application, the thin film layers and the substrate will need to be transparent [97], rendering this method unfeasible; however, this method is non-contact and non-destructive, which could potentially be applied to other coating/substrate material combinations for in-line measurement.

### 4.7. Self-Mixing Interferometry (SMI)

Self-mixing interferometry is a non-contact optical method which has been employed for the measurement of refractive indices and thickness measurement of optical components [98]. This method operates on similar principles to standard interferometry, using the measurement of optical path length; however, this method utilizes the phase difference of a back scattered beam without a beam splitter and reference measurement [99]. The self-mixing interferometer emits a collimated beam from a laser and is passed through a transparent sample. Upon reflection at a second photodiode [100], some of the reflected light is re-entered into the laser cavity where the weak signal interacts with the incident beam. This results in modulation of amplitude and frequency of the laser field where the driver is the optical path length. This method provides non-contact, non-destructive measurement and has proven research at the micron thickness level [98,99,100]. For this application, this method is unfeasible as SMI requires optical transmission through both the coating and substrate but could potentially be applied to other transparent coating/substrate material combination applications for in-line measurement.

### 4.8. Chromatic Confocal Microscopy (CCM)

The CCM method is a non-destructive and non-contact optical technique that has been researched for film thickness measurement for transparent and non-transparent films [101,102]. An incident white light source is focused into its spectrum of wavelengths for varying distances to the sample, and in terms of film thickness measurement, two peaks relational to the wavelengths and distance to the sample are calculated, the difference between these peaks would result in the optical thickness of the measured material. When the optical properties of the material are known, the physical thickness can be calculated via the dispersion properties as a function of wavelength in terms of the sample optical properties [102]. This method has been reported to have micron thickness resolution, and feasible for in-line implementation [102]. There are several researched variants of the CCM technique, one such variant is Chromatic Confocal Spectral Interferometry (CCSI), which combines the operating principles of CCM and optical interferometry for increased resolution [103]. Although current literature reports only micron thickness resolution, it is assessed that this method may be developed further, rendering this method potentially feasible for this application.

### 4.9. Infrared Thermography

Infrared imaging has been previously used to measure the coating thickness of paints on steel substrates with a coating thickness at the micron level [104]. The principle of operation is to apply external heating of a sample and measure the thermal radiation via a high-resolution thermal camera within the IR spectrum [104]. The benefits of this measurement method are that it can image a large spatial area concurrently, allowing for the monitoring of the coating uniformity real time. The underlying principle of this method is the correlation between the heat transfer of each coating layer with the layer thickness [104]. As most elements have differing heat transfer properties, this method makes it suitable to measure many combinations of substrate and coating materials. Some of the key requirements for this method is the introduction of an external heating source, a high-resolution thermal IR detector capable of nanometer resolution and the heat transfer properties between coating layers and substrate are diverse. This method is non-contact, non-destructive and can potentially be used for a vast number of coatings and substrates with high-speed detection [104]. In terms for this application, for coating thickness at the nanometer scale, the detection elements to measure the full width of the electroplated product would require great expense, not to mention, the heating element may also cause complications to the production process dependent on the amount of heating required to trigger accurate detection. One variant of this technique discussed briefly previously was a thermoreflectance imaging approach [69]. This technique operates on the reverse process of the Seebeck effect, known as the Peltier effect. By exciting the sample under test with an alternating current supply, this in turn would generate an oscillating temperature field in the sample, which could then be detected using a CCD. This technique has been used in the semi-conductor industry [69], providing a topographical image of the heat propagation at sub-micron thickness resolution.

### 4.10. Overview of Potential In-Line Coating Thickness Methodologies

Table 3 summarizes the potential and novel in-line coating thickness test methods that have high potential for this research application. Nine potential in-line coating thickness measurement methodologies are reviewed for this steel packaging application, these are thermoelectric magnetic method (C1), THz-TDS (C2), Optical reflectometry (C3), Optical interferometry (C4), Optical ellipsometry (C5), SBS (C6), SMI (C7), CCM (C8), and infrared thermography (C9). From a commercial product survey, the thermoelectric magnetic, SBS, and SMI methodologies were not found to have a specific product for a coating thickness application. It must also be noted that at this current time, only three methods have been found through research for the capability to measure coatings of nanometer in thickness, these are optical reflectometry, ellipsometry, and interferometry. There are several variants of these three optical methods as highlighted previously in this review paper, and there are also commercial systems available for these methodologies; however, none of these systems specify their unique ability to be applied to this steel packaging application, specifically the metallic chromium layer. It was interesting to find through the reflectometry commercial product analysis [105] that it states that reflectometry and ellipsometry have the capability to measure metallic coatings of under 50 nm in thickness, which directly relates to the outlined literature, which reinforces the high potential of these methodologies in particular for further research and development or this steel packaging application, as we are intending investigate metallic chromium coatings under these thickness limits. In terms of the detection area of these methodologies, these are highly dependent on the system construction, which can be clearly noted from the commercial product survey and the appropriate citations. The interferometry method highlighted from the commercial product survey is based on Optical Coherence Tomography (OCT), where the method provides similar output results to AFM, non-destructively, and without contact with the sample under test.

From this review table, there are three clear methodologies to pursue for future research into this steel packaging application, these are optical reflectometry, optical ellipsometry and optical interferometry. Taking into consideration the system complexity, cost and potential, reflectometry and its variants are highlighted as the focus of future research, followed by interferometry and ellipsometry.

## 5. Conclusions

This review has attempted to identify several potential in-line coating thickness measurement methods that could be applied to measure nanometer chrome coatings on a steel substrate. Each method outlined within this paper has been categorized into their potential for: continuous, non-contact, non-destructive, in-line measurement; at speed of sub 50 nm coating thickness measurement of applicable substrate/coating material combinations. From this interim investigation there are three main optical methods which have been assessed as highly feasible for this research application, optical reflectometry, ellipsometry, and interferometry. There are also other novel methodologies which may satisfy this application requirements such as a thermoelectric method with a magnetic readout approach, THz-TDS, CCM, and infrared thermography; however, there is very little research regarding these methods for nano-scale precision. The X-ray methods outlined such as XRR and XRF are also highly feasible for this application; however, this research is based upon finding a novel approach from the current coating thickness test methods implemented at the industrial partner. There is an abundance of research in the literature suitable to establish a novel method to measure metallic coating thickness for steel packaging applications. All three optical methodologies identified have also been reviewed for potential in-line monitoring for other applications [111], and it can clearly be seen through the literature that optical techniques are being highlighted as the successors for potential in-line monitoring of nanometric coatings. For the optical methods outlined, there is the potential to: create a low-cost system; implement this low-cost system at multiple points at within a production line; multiplex measurements across the width of a production line to ensure full width coverage; and to provide redundancy to currently used XRF-based systems that are unable to differentiate between metallic and oxide chromium layers. In terms of the applicable optical methods, there are several variants that have been researched, developed, and implemented in other industries. Consequently, this research aims to develop these techniques, or variants thereof, for steel packaging applications with metallic sub-50 nm coating thicknesses. In addition to this review paper, further research will be conducted into the three optical methods highlighted via simulations and experimental research for this application based on the requirements set out within this paper, with emphasis placed on other significant factors that may hinder the feasibility of these methods such as the effect of illumination wavelength, effect of incident angle, effect of chrome oxide layer within the sample, effect of differing substrate chemistry, effect of substrate surface roughness resultant from the steel rolling process, and the effect of vibrational parameters on a continuous production line.

## Figures and Tables

**Table 1:** Overview of traditional coating thickness methodologies.

Methodology	A1	A2	A3	A4	A5	A6	A7
Thickness ranges	1 nm–50 µm	1–800 µm	1–10,000 µm	1–10,000 µm	0.5 nm–10 µm	1 nm–1 µm	10–7500 µm
Measurement accuracy (%)	0.1–0.5	0–5	0.1–0.7	1–3	0.2–0.5	0.33–0.65	0.53–0.7
Multi-layer	✓	✓	X	X	✓	✓	✓
Sampling rate	1–500 m	0.5–15 s	0.6–1 s	0.6–1 s	0.5–100 s	1 s–60 m	0.5–2.5 s
Detection area (ø)	1.5–3.2 mm	0.63–20 mm	5–8 mm	5–8 mm	0.1–15 mm	2 mm	5 mm
Commercial availability	[47]	[48,49]	[50]	[50]	[51]	[52]	[53]
Typical cost (GBP)	>3000	Unspecified	>1000	>1000	>35,000	>30,000	>1000
Non-contact	X	✓	✓	X	✓	✓	X
Non-destructive	X	✓	✓	✓	✓	✓	✓
Materials	✓	X	X	✓	✓	✓	X

**Table 2:** Overview of offline coating thickness methodologies.

Methodology	B1	B2	B3	B4
Thickness ranges	0.5–20 nm	0.1 nm–2 µm	0.1–100 nm	1 nm–50 µm
Measurement accuracy (%)	0.05	0.2	2–5	<5
Multi-layer	✓	✓	✓	✓
Sampling rate	1 m–4 h	1–5 m	5 m–1 h	3 s–12 m
Detection area (ø)	10 µm–5 mm	50 nm–1 cm	10–100 µm	Unspecified
Commercial availability	[63]	[64]	[65]	[66]
Typical cost (GBP)	>200,000	>50,000	>20,000	Unspecified
Non-contact	✓	✓	X	X
Non-destructive	✓	✓	✓	X
Materials	✓	✓	✓	✓

**Table 3:** Overview of potential in-line coating thickness methodologies.

Methodology	C1	C2	C3	C4	C5	C6	C7	C8	C9
Thickness ranges	1–200 µm	300–1400 µm	0.5 nm–3 mm	0.1 nm–10 µm	0.5 nm–1 mm	>1 µm	>1 µm	>1 µm	>1 µm
Measurement accuracy (%)	10	0.43	0.1–0.2	<1	0.1	Unspecified	Unspecified	<3%	Unspecified
Multi-layer	Unspecified	✓	✓	✓	✓	✓	✓	✓	✓
Sampling frequency	0.1–4 s	>50 ms	0.1–5 s	<3 s	0.1–300 s	Unspecified	Unspecified	<1 s	<1 s
Detection area (ø)	Unspecified	Unspecified	50 µm–1 mm	Unspecified	50 µm–1 mm	Unspecified	Unspecified	Unspecified	Unspecified
Commercial availability	Unspecified	[106]	[105]	[107]	[108]	Unspecified	Unspecified	[109]	[110]
Typical cost (GBP)	Unspecified	Unspecified	>13,000	>45,000	>40,000	Unspecified	Unspecified	>10,000	>10,000
Non-contact	✓	✓	✓	✓	✓	✓	✓	✓	✓
Non-destructive	✓	✓	✓	✓	✓	✓	✓	✓	✓
Materials	✓	✓	✓	✓	✓	X	X	✓	✓
